# Variation of Red Blood Cell Distribution Width and Mean Platelet Volume after Moderate Endurance Exercise

**DOI:** 10.1155/2014/192173

**Published:** 2014-08-13

**Authors:** Giuseppe Lippi, Gian Luca Salvagno, Elisa Danese, Cantor Tarperi, Gian Cesare Guidi, Federico Schena

**Affiliations:** ^1^Laboratory of Clinical Chemistry and Hematology, Academic Hospital of Parma, Via Gramsci 14, 43126 Parma, Italy; ^2^Laboratory of Clinical Biochemistry, Department of Life and Reproduction Sciences, University of Verona, Via delle Menegone, 37100 Verona, Italy; ^3^Department of Neurological, Neuropsychological, Morphological and Movement Sciences, University of Verona, Via delle Menegone, 37100 Verona, Italy

## Abstract

Although physical exercise strongly influences several laboratory parameters, data about the hematological changes after medium distance running are scarce. We studied 31 middle-trained athletes (mean training regimen 217 ± 32 min/week) who performed a 21.1 km, half-marathon run. Blood samples were collected before the run, at the end, and 3 and 20 hours thereafter. The complete blood count was performed on Advia 2120 and included red blood cell (RBC), reticulocyte, and platelet counts; hemoglobin; mean corpuscular volume (MCV); mean corpuscular hemoglobin (MCH); reticulocyte haemoglobin content (Ret CHR); RBC distribution width (RDW), mean platelet volume (MPV). No significant variations were observed for MCH and Ret CHR. The RBC, reticulocyte, and hemoglobin values modestly decreased after the run. The MCV significantly increased at the end of running but returned to baseline 3 hours thereafter. The RDW constantly increased, reaching a peak 20 hours after the run. The platelet count and MPV both increased after the run and returned to baseline 3 hours thereafter. These results may have implications for definition of reference ranges and antidoping testing, and may also contribute to explaining the relationship between endurance exercise and mortality, since previous studies reported that RDW and MPV may be significantly associated with cardiovascular disease.

## 1. Introduction

Physical exercise is an important preanalytical variable, which strongly influences several biological and metabolic pathways. These remarkable variations are often reflected by concomitant changes of a number of laboratory parameters [1]. The accurate identification of such paraphysiologic variations is pivotal in medicine and sports not only to prevent misinterpretation of data and hence to define the real state of health and fitness of the athletes but also to detect the potential use of unfair doping practices and to assist sport physicians in follow-up of athletic injuries [1].

Some previous studies have investigated the changes of red blood cell (RBC) and platelet biology that may be induced by endurance exercise [2–6], but the outcome of these trails was often controversial due to the heterogeneity of study populations (trained or untrained athletes), settings (sea level or altitude), types of sport (sprint, endurance, or mixed), and volumes and intensities of the physical effort (short, medium, or long term performance). Among the various sports disciplines, medium distance running (i.e., from 10 to 21 km) is the most practiced worldwide at both recreational or competitive levels [7], since it provides a good balance between time spent in running and job or personal life and does not require the level of fitness and training of marathon or ultramarathon running [8]. It has been reported, however, that the risk of cardiac complications after medium and long distance running is not meaningless, with an overall incidence of cardiac arrest of 0.37 and 0.19 per 100,000 runner hours in marathon runners and half-marathon runners, respectively [9].

Data about the hematological changes after medium distance running are scarce to the best of our knowledge. Even more importantly, the changes in RBC distribution width (RDW) and mean platelet volume (MPV), which are two emerging biomarkers of cardiovascular disease and overall mortality [10, 11], are lacking. As such, we planned a prospective study to assess the changes of some parameters of the complete blood cell count (CBC) in athletes performing moderate endurance exercise in order to establish whether the increased cardiovascular problems that are occasionally observed after this kind of exercise may be mirrored by variation of either RDW or MPV.

## 2. Materials and Methods

The study population consisted in 31 middle-trained athletes (11 females and 20 males; mean age 44 ± 7 years), who performed a 21.1 km, half-marathon run. The athletes were recruited from a team of nonprofessional, amateur runners regularly involved in recreational running, with mean training regimen of 217 ± 32 min/week and maximal oxygen uptake of 51 ± 5 mL/kg/min. No exclusion criteria were applied. The distance was run on a hilly and demanding route in the town of Verona, Italy (197 m altitude gap, with inclines averaging 1.8% and peaks up to 7%), in a partially sunny day with temperatures between 14 and 17°C and humidity between 60 and 80%. Blood samples were collected in primary blood tubes containing K_2_EDTA (Terumo Europe N.V., Leuven, Belgium) immediately before the run and at the end of the trial, as well as 3 and 20 hours thereafter. All samples were immediately transported to the clinical chemistry laboratory of the Academic Hospital of Verona under controlled conditions of temperature and humidity. The CBC was performed on Advia 2120 (Siemens Healthcare Diagnostics, Tarrytown NY, USA) and included RBC, reticulocyte, and platelet counts; hemoglobin; mean corpuscular volume (MCV); mean corpuscular hemoglobin (MCH); reticulocyte haemoglobin content (Ret CHR); RDW; MPV. Results of measurements were expressed as median and interquartile range (IQR) and differences were analyzed with Wilcoxon test for paired samples, using Analyse-it (Analyse-it Software Ltd, Leeds, UK). All subjects gave an informed consent for being enrolled in this study, which was approved by the local academic ethical committee and performed in accordance with the Helsinki Declaration of 1975.

## 3. Results

The results of this study are shown in Table 1. No significant variations were observed for MCH and Ret CHR throughout the study period. The number of RBC and the hemoglobin values decreased significantly immediately after the run and remained significantly decreased compared to the baseline up to 20 hours thereafter. A similar trend was observed for the absolute reticulocyte count. The MCV was found to be significantly increased at the end of the run, but values returned similar to the baseline 3 hours thereafter. The RDW exhibited a remarkable trend towards increased values, reaching the peak 20 hours after the end of the run (median increase, 2.2%; IQR, 0.8–3.3%). Both the platelet count and the MPV significantly increased after the end of the run (median increase of MPV, 5.7%; IQR, 0.6–9.3%), whereas the values of both parameters returned to values similar to the baseline 3 hours thereafter. The odds ratio (OR) for an increased RDW value (i.e., >13.8%) was 3.7 (95% CI, 1.03–13.4; *P* = 0.044) 20 hours after the end of the run. Similarly, the OR for an increased MPV value (i.e., >8.5 fL) was 10.4 (95% CI, 1.22–89.5; *P* = 0.03) 3 hours after the end of the half-marathon run.

## 4. Discussion

The results of this prospective study, which demonstrated for the first time that both RDW and MPV values may significantly vary in response to moderate endurance exercise, may have at least three meaningful implications. The first important issue is that the conventional reference ranges of some parameters of the CBC, including those of the RDW and MPV, may only be applicable in subjects at rest because all the other parameters tested in this study exhibited significant variations throughout the observational period. It is noteworthy that although the maximum percentage variation of the RDW remained lower than the within-subject biologic variation (i.e., 2.2% versus ±3.5%), the percentage increase of MPV largely exceeded the within-subject biologic variation (i.e., 5.7% versus ±4.3%) and should hence be considered clinically significant [12]. Interestingly, we also observed that the hemoglobin values marginally but significantly decreased immediately after the run, and persisted significantly reduced up to 20 hours thereafter. This is an original finding that was not observed in a previous similar investigation [13] but has minor clinical significance, since the maximum percentage variation observed in our study (i.e., −3.2%) was very close to the within-subject biologic variation (±3%) [12].

Another important issue is the potential impact of these variations on antidoping testing. The athlete biological passport (ABP) is a reliable strategy to monitor selected biological variables over time that indirectly reveal the effects of doping and includes a large number of hematological variables [14]. More specifically, the so-called “adaptative model” currently used by the World Anti-Doping Agency (WADA) is based on an algorithm integrating hemoglobin, RBC count, reticulocytes count, MCV, and MCH. According to our data, all these variables except MCH were variably modified by moderate endurance exercise, and this should hence be clearly acknowledged when interpreting results of both the hematological module and the abnormal blood profile score (ABPS) [15].

Some conclusions can also be made about the impact of a half-marathon run on the cardiovascular risk. Several lines of evidence now attest that a high RDW is associated with an increased risk of mortality and morbidity in patients with heart disease [16], as well as in the general population [17, 18]. Similarly, an increased MPV conventionally reflects platelet hyperreactivity [19], that is, a leading player in the complex pathogenesis of cardiovascular disorders [20]. This aspect has been confirmed in a recent meta-analysis showing that elevated MPV is associated with acute myocardial infarction, mortality following myocardial infarction, and restenosis after coronary angioplasty [21].

Although it has not been definitely elucidated whether anisocytosis and a larger platelet volume are risk factors or simple biomarkers of disease, the observation that RDW and especially MPV consistently increase after a moderate endurance effort reflects a clear perturbation of RBC and platelet biology. This is noteworthy considering that, according to recent statistics, the overall deaths per 100,000 marathon finishers were estimated at 0.75 (95% confidence interval [CI], 0.38–1.13) and, even more interestingly, myocardial infarction or atherosclerotic heart disease accounted for the vast majority of deaths (i.e., >90%) [22]. The results of this study should hence represent a reliable basis to further define whether anisocytosis and larger platelet volume may effectively contribute to explanation of the relationship between endurance exercise and mortality, especially in subjects at higher risk of cardiovascular disease.

## Figures and Tables

**Table 1:** Haematological changes in middle-trained athletes undergoing a half-marathon run.

	Baseline	Post run	After 3 h	After 20 h
RBC (10^12^/L)	4.9 (4.6–5.1)	4.8^†^ (4.5–5.1)	4.6^‡^ (4.3–4.9)	4.7^‡^ (4.4–4.9)
Hemoglobin (g/L)	149 (145–155)	148^†^ (140–155)	142^‡^ (136–148)	144^‡^ (139–150)
MCV (fL)	94.9 (91.9–96.3)	95.3^†^ (93.5–97.3)	94.1 (92.3–95.8)	94.9 (91.5–97.1)
MCH (pg)	31.0 (30.0–31.1)	31.5 (30.0–32.0)	31.0 (30.3–32.8)	30.5 (30.0–32.0)
RDW (%)	13.3 (13.1–13.5)	13.4^†^ (13.1–13.6)	13.4^‡^ (13.2–13.6)	13.5^‡^ (13.3–13.8)
Reticulocytes (10^9^/L)	60.5 (49.5–72.5)	60.4 (49.6–74.2)	58.0^‡^ (46.7–69.3)	59.7^‡^ (46.9–65.9)
Ret CHR (pg)	31.0 (31.0–32.0)	31.0 (31.0–32.0)	31.0 (31.0–32.0)	31.0 (31.0–32.0)
Platelets (10^9^/L)	255 (216–298)	311^‡^ (264–361)	263 (219–290)	255 (212–290)
MPV (fL)	9.2 (8.6–9.9)	9.5^‡^ (9.1–10.2)	9.1 (8.1–9.4)	9.2 (8.7–9.7)

^†^ *P* < 0.05 compared to baseline; ^‡^ *P* < 0.01 compared to baseline.

MCH: mean corpuscular hemoglobin; MCV: mean corpuscular volume; MPV: mean platelet volume; RBC: red blood cell; RDW: RBC distribution width; Ret CHR: reticulocyte hemoglobin concentration.

## Abbreviations

CBC: Complete blood cell count
IQR: Interquartile range
MCH: Mean corpuscular hemoglobin
MCV: Mean corpuscular volume
MPV: Mean platelet volume
RBC: Red blood cell
RDW: RBC distribution width
Ret CHR: Reticulocyte hemoglobin concentration.
